# MCM proteins are up-regulated in placentas of women with reduced insulin sensitivity

**DOI:** 10.1042/BSR20240430

**Published:** 2024-10-08

**Authors:** Julia Bandres-Meriz, Marta Inmaculada Sanz-Cuadrado, Irene Hurtado de Mendoza, Alejandro Majali-Martinez, Sophie Elisabeth Honeder, Tereza Cindrova-Davies, Ruth Birner-Gruenberger, Louise Torp Dalgaard, Gernot Desoye

**Affiliations:** 1 Department of Obstetrics and Gynaecology, Medical University of Graz, Graz, Austria; 2 Departamento de Medicina, Facultad de Ciencias Biomédicas y de la Salud. Universidad Europea de Madrid, Madrid, Spain; 3 Institute of Pathology, Diagnostic and Research Center for Molecular Biomedicine, Medical University of Graz, Graz, Austria; 4 Institute of Chemical Technologies and Analytics, Technische Universität Wien, Vienna, Austria; 5 Centre for Trophoblast Research, Department of Physiology, Development and Neuroscience, University of Cambridge, Cambridge, U.K.; 6 Department of Science and Environment, Roskilde University, Roskilde, Denmark

**Keywords:** insulin resistance, obesity, placenta

## Abstract

In the first trimester of pregnancy the human placenta grows rapidly, making it sensitive to changes in the intrauterine environment. To test whether exposure to an environment *in utero* often associated with obesity modifies placental proteome and function, we performed untargeted proteomics (LC-MS/MS) in placentas from 19 women (gestational age 35–48 days, i.e. 5^+0^–6^+6^ weeks). Maternal clinical traits (body mass index, leptin, glucose, C-peptide and insulin sensitivity) and gestational age were recorded. DNA replication and cell cycle pathways were enriched in the proteome of placentas of women with low maternal insulin sensitivity. Driving these pathways were the minichromosome maintenance (MCM) proteins MCM2, MCM3, MCM4, MCM5, MCM6 and MCM7 (MCM-complex). These proteins are part of the pre-replicative complex and participate in DNA damage repair. Indeed, MCM6 and γH2AX (DNA-damage marker) protein levels correlated in first trimester placental tissue (*r* = 0.514, *P*<0.01). MCM6 and γH2AX co-localized to nuclei of villous cytotrophoblast cells, the proliferative cell type of the placenta, suggesting increased DNA damage in this cell type. To mimic key features of the intrauterine obesogenic environment, a first trimester trophoblast cell line, i.e., ACH-3P, was exposed to high insulin (10 nM) or low oxygen tension (2.5% O_2_). There was a significant correlation between MCM6 and γH2AX protein levels, but these were independent of insulin or oxygen exposure. These findings show that chronic exposure *in utero* to reduced maternal insulin sensitivity during early pregnancy induces changes in the early first trimester placental proteome. Pathways related to DNA replication, cell cycle and DNA damage repair appear especially sensitive to such an *in utero* environment.

## Introduction

The periconceptional and early pregnancy period are critical for fertilization, placentation and embryogenesis [1,2]. These processes require physiological adaptations in the mother and are sensitive to changes in the intrauterine environment [3]. The rapid growth of the human placenta in the first trimester makes it particularly sensitive to perturbations in the intrauterine environment [4,5].

Maternal overweight and obesity, clinically defined as body mass index (BMI) 25.0–29.9 kg/m^2^ and ≥30 kg/m^2^, respectively, associate with metabolic changes in the intrauterine environment that are present already in the first trimester [6,7]. It affects embryonic development and is associated with reduced early fetal growth [8]. This may compromise further fetal development and increase the risk for metabolic diseases later in life. Indeed, offspring born from pregnancies complicated by obesity are at higher risk of developing obesity and metabolic diseases in adulthood [9].

In the first trimester of pregnancy, embryonic and fetal growth associate with placental volume and utero-placental vascular volume [10]. Lower volumes in pregnancies complicated by obesity suggest reduced growth not only of the fetus [8] but also of the placenta. In a previous study, we found that maternal obesity alters cell cycle and DNA-damage checkpoint regulators (i.e., breast cancer 1: BRCA1; checkpoint kinase 1: Chk1; checkpoint kinase 2: Chk2; suppressor of mothers against decapentaplegic 2/3: SMAD2/3; cell division cycle 25C: CDC25C) in the placenta [11], suggesting increased DNA damage and/or impaired DNA damage repair. However, neither the metabolic traits associated with maternal obesity that impair DNA stability nor the contributing molecular species have been identified.

In the present study, we hypothesized that exposure to an environment often associated with obesity affects placental development very early in pregnancy (week 5^+0^–6^+6^ based on the last menstrual period, LMP) by altering the placental proteome. Specifically, we aimed to identify key players in the biological processes severely affected by placental exposure to a metabolically adverse environment

To this end, we first analysed the proteome of 19 first trimester placentas (gestational age 5^+0^–6^+6^ weeks) of women with a wide BMI range (19.7–32.4 kg/m^2^) kg/m^2^) to cover metabolic variations. Then we identified the clinical traits that associated with the proteome signature. These analyses lead to the further focus on up-regulated key proteins, e.g., minichromosome maintenance (MCM) 6, and we studied their cellular location in first trimester placenta sections. Finally, we conducted *in vitro* experiments to examine the potential effect of key traits dysregulated in the obese intrauterine environment during the first trimester of pregnancy on the identified proteins. These *in vitro* experiments were carried out using an established first trimester trophoblast cell line.

## Materials and methods

### Study participants and placental collection

The study was conducted in accordance with the World Medical Association Declaration of Helsinki and approved by the ethical committee of the Medical University of Graz (no. 29-095 ex 16/17, December 23, 2016 and 31-094 ex 18/19, March 1, 2019). All subjects provided written informed consent. Patient details were limited to those listed in Table 1 to protect patient privacy as a requirement of the ethics approval. Women undergoing a voluntary pregnancy termination (*n*=35) were recruited. Allocation of samples to experiments is shown in Supplementary Figure S1. Inclusion criteria were a singleton pregnancy and no comorbidities, e.g., pre-existing diabetes mellitus, hypertension. Exclusion criterion was maternal age <18 years. Gestational age (days) was calculated based on the last menstrual period (LMP) and confirmed by ultrasound measurement of the crown-rump length. Participants’ height (m) and weight (kg) were measured on the day of pregnancy termination and used to calculate the BMI (kg/m^2^). Placental tissues were collected during surgical pregnancy termination, washed with phosphate buffered saline (PBS, Sigma-Aldrich, St. Louis, MO, U.S.A.) and snap-frozen in liquid nitrogen. Maternal blood was collected after overnight fast and used to measure concentrations of leptin (ng/ml), glucose (mmol/l) and C-peptide (pmol/l), as previously described [6]. The Homeostatic model assessment of insulin sensitivity index (IS_HOMA_) was calculated using fasting glucose and C-peptide values [12].

**Table 1:** Maternal clinical traits of the cohort used for proteomic analysis

	Discovery cohort (*n*=19)
	Median; IQR
Gestational age (days)	36 (35–46)
Maternal age (years)	32 (28–39)
BMI (kg/m^2^)	21.8 (19.7–29.0)
Leptin (ng/ml)	13.6 (6.1–20.6)
Glucose (mmol/l)	5.4 (4.7–6.0)
C-peptide (pmol/l)	421.2 (306.4–599.2)
IS_HOMA_ index	0.61 (0.37–0.76)

Maternal clinical traits (median, IQR) of the women whose placental tissue was subjected to untargeted proteomics analysis (*n*=19).

Abbreviations: BMI, body mass index; IS_HOMA_, homeostatic model assessment of insulin sensitivity; IQR, interquartile range.

### Untargeted proteomics in placental tissue

The proteome of 19 first trimester placentas (gestational age week 5^+0^–6^+6^ LMP) was analyzed with untargeted proteomics (LC-MS/MS) as previously described [13]. Placenta tissue was thawed and suspended in Dulbecco’s Phosphate Buffered Saline (PBS) (Sigma-Aldrich, Burlington, MA, U.S.A.). After 1 h incubation at 37°C, the tissue was lysed by sonication (10 s, 70% amplitude) with a Sonoplus mini20 hand sonicator. Protein content was quantified using the bicinchoninic acid assay method (Pierce™ BCA Protein Assay kit; Thermo Fisher Scientific, Waltham, MA, U.S.A.) [14]. The lysate was brought to 100 µl PBS containing 50 µg protein. For reduction and alkylation, 10 mM tris (2-carboxyethyl)-phosphine hydrochloride (TCEP) (Sigma-Aldrich) and 40 mM 2-chloracetamide (Sigma-Aldrich) were added followed by heating to 95°C for 10 min. Acetone precipitation was performed overnight (–20°C). After centrifugation (14,000 × ***g***; 10 min; 4°C), the protein pellet was dissolved in 25% trifluoroethanol (TFE) in 100 mM Tris-HCl (pH 8.5), which was subsequently diluted to 10% TFE with 100 mM ammonium bicarbonate. Proteins were pre-digested for 2h using 1:100 (w/w) LysC (Promega, Madison, WI, U.S.A.) at 37°C and further digested overnight with 1:100 (w/w) sequencing-grade modified trypsin (Promega) at 37°C. The resulting peptide solution was brought to 1% trifluoroacetic acid (TFA). Peptide solution containing 4 g protein was used for desalting with Styrene Divinylbenzene-Reversed Phase Sulfonate (SDB-RPS) stage tips (Empore, CDS analytical, Oxford, PA, U.S.A.). After desalting, samples were dried in a vacuum concentrator centrifuge and dissolved in 0.1% formic acid (FA), 2% acetonitrile (ACN) in water.

Samples were analyzed by nano-HPLC (Dionex Ultimate 3000, Thermo Fisher Scientific) equipped with an Aurora Series Emitter nanocolumn with CSI fitting (C18, 1.6 µm, 120 Å, 250 × 0.075 mm). Peptides were separated at 35°C at a flow rate of 300 nl/min. Solvent A (0.1% FA in water) and solvent B (0.1% FA in ACN) were used in a 133 min chromatography gradient from 2% B up to 95% B. Chromatography was coupled to a Bruker maXis II ETD Q-TOF MS (Bruker, Billerica, MA, U.S.A.) operated with nano-ESI source in positive mode.

For label-free quantitation, MaxQuant software (version 1.6.0.16) with the integrated peptide search engine Andromeda was employed [15]. The match-between-runs feature of MaxQuant was enabled with a retention window of 0.7 min and an alignment window of 20 min. For the database search, the false discovery rate (FDR) was set to 1% for both peptides and proteins [15]. Subsequent steps included unsupervised principal component analysis and two-sided unpaired *t-*test with permutation-based FDR control at a level of 5%.

Mass spectrometry proteomics data was deposited to the ProteomeXchange Consortium via the PRIDE [16] partner repository with the data set identifier PXD056380.

### Bioinformatics

Downstream data analysis was performed in Perseus (version 1.6.10.43) [17]. Filtering criterion was set to proteins with at least 9 valid values (>0) in at least one group (IS_HOMA_ ≤0.61; IS_HOMA_ >0.61). This was followed by imputation of missing values from normal distribution with regards to the total data matrix. Unsupervised principal component analysis was used to identify clusters in the data. The IS_HOMA_ cut-off was based on the median IS_HOMA_ of the cohort (median IS_HOMA_ = 0.61). Two-sided unpaired T-test (FDR = 0.05) was used to identify proteins that differ between placentas of women with high and low insulin sensitivity. All the proteins in the low IS_HOMA_ group, which were enriched with a *P*<0.05 (compared with the high IS_HOMA_ group), were selected for building a protein–protein interaction network and functional enrichment analysis in String, version 11.5 [18]. The predefined *Homo sapiens* library in String uses Ensembl ver. 99 [19] and served as background. The statistically significant enriched terms, for each type of analysis i.e., pathway, biological process, molecular function, cellular component, were selected (based on Benjamini and Hochberg false discovery rate *P*-values).

### Cell culture

The first trimester trophoblast cell line ACH-3P was cultured in Ham’s F12 medium with glutamine (Biowest, Nuaillé, FR) supplemented with 10% (v/v) defined fetal bovine serum (FBS, Cytiva, Marlborough, MA, U.S.A.) and 1% (v/v) penicillin/streptomycin (Thermo Fisher Scientific). Cells were used within two passages to avoid phenotypic drift and were visually monitored. The cells (2.5 × 10^4^ cells/well in 6-well plates) were transferred to a hypoxic workstation (Xvivo System Model X3, BioSpherix, Redfield, NY, U.S.A.) and allowed to accommodate to the respective oxygen tension for 48 h. All experiments were performed at 6.5% O_2_, except the oxygen challenge experiment in which cells were cultured at 2.5%, 6.5% or 21% O_2_. Cell viability (cell morphology and colony formation) was visually examined using a microscope (EVOS Cell Imaging System, Thermo Fisher Scientific) integrated in the hypoxia chamber.

#### Titration of DNA damage induction

After 72 h in culture, ACH-3P cells were challenged for 1 h with 1, 5 or 10 µM etoposide (Cayman Chemicals, Ann Arbor, MI, U.S.A.) or DMSO (vehicle) (Sigma-Aldrich). Subsequently, cells were collected for gene expression and protein analyses.

#### DNA damage induction and recovery

Cells were maintained in culture for 72 h and thereafter challenged with 10 µM etoposide or DMSO (vehicle) for 1 h. Medium was aspirated and the cells were allowed to recover for 3 or 24 h in fresh medium (Ham’s F12, 10% FBS, 1% penicillin/streptomycin), followed by cell collection for subsequent gene expression and protein analyses.

To investigate γH2AX and MCM6 co-localization in response to different DNA damage inducers, ACH-3P cells were cultured in 4-well chamber slides (1 × 10^4^ cells/well) for 48 h. Thereafter, cells were treated for 1 h with 10 µM etoposide or 10 µM bleomycin (Sigma-Aldrich) (DMSO or F12 medium as respective vehicle controls) and further allowed to recover in fresh medium (Ham’s F12, 10% FBS, 1% penicillin/streptomycin) for 1h. Thereafter, ACH-3P cells were immunostained and γH2AX and MCM6 co-localization assessed.

#### Insulin treatment

After 48 h in culture (6.5% O_2_), the medium was replaced by low serum medium (Ham’s F12, 2% FCS, 1% penicillin/streptomycin) for 24 h to reduce insulin in the serum (final insulin concentration: 1.4 × 10^−4^ nM). Thereafter, cells were cultured in the absence or presence of 10nM insulin (Calbiochem, San Diego, CA, U.S.A.) for further 24 or 48 h and collected for gene expression and protein analyses.

#### Oxygen challenge

To closely mimic *in vivo* physiology, cells were cultured for 48 h at 2.5% O_2_ (physiological oxygen tension until approximately week 8 of pregnancy) [20]. After 48 h, cell were maintained at 2.5% O_2_ or transferred to 6.5% O_2_ or 21% O_2_ for additional 48 h, respectively.

### RNA isolation and reverse transcription polymerase chain reaction (RT-qPCR)

Total RNA was isolated using the miRNeasy Tissue/Cells Advanced Micro Kit (Qiagen, Hilden, DE) according to manufacturer’s instructions. The methods uses guanidine-based tissue and cell lysis followed by silica-membrane-based purification of total RNA. RNA was reverse-transcribed using the Luna Script RT SuperMix Kit (New England BioLabs, Ipswich, MA, U.S.A.). TaqMan universal PCR master mix (Life Technologies, Carlsbad, CA, U.S.A.) and TagMan gene expression assays (MCM6: Hs00195504_m1; MCM3: Hs00172459_m1; Thermo Fisher Scientific) were used to assess MCM6 and MCM3 mRNA expression by RT-qPCR in a CFX384 RT-qPCR detection system (Bio-Rad Laboratories, Hercules, CA, U.S.A.). Cycle threshold (*C*t) values were automatically generated by Bio-Rad CFX Manager 3.1 software (Bio-Rad Laboratories, Hercules, CA, U.S.A.). Δ*C*t values were calculated using the housekeeping genes TATA-binding protein (TBP: Hs03929085_g1, Thermo Fischer Scientific) and peptidyl-prolyl isomerase (PPIA, Hs04194521_s1, Thermo Fisher Scientific). For graphical representation, the results were normalized to the reference category (insulin experiments: vehicle control; oxygen experiments: 2.5% O_2_), using the 2−ΔΔ*C*t method.

### Protein isolation and Western blotting

Placental tissue and ACH-3P cells were resuspended in RIPA buffer (Sigma-Aldrich) containing protease (Roche, Mannheim, DE) and phosphoprotease inhibitors (MedChemExpress, Princeton, NJ, U.S.A.). Placental tissue was homogenized in a MagNa Lyser device (Roche). Protein concentration was quantified using the bicinchoninic acid assay (Thermo Fisher Scientific). Protein lysates were mixed with Laemmli buffer 2x (Sigma-Aldrich) and denatured at 95°C for 5 min. Equal amounts of total protein (10 µg/well for tissue lysates and 5 µg/well for cell lysates) were loaded on to 4–20% SDS-PAGE gels (BioRad Technologies) and resolved at 120 V for 75 min. Proteins were transferred to a nitrocellulose membrane (BioRad Technologies) and stained with Ponceau S (Thermo Fisher Scientific). Non-specific binding sites were blocked for 1 h with 5% (w/v) non-fat dry milk (BioRad Laboratories) in tris-borate-EDTA (TBE) + 0.1% (v/v) Tween 20 (Sigma-Aldrich). After blocking, the membranes were incubated with primary antibodies against MCM6 (1:500 Abcam, cat# ab201683, Cambridge, U.K.); γH2AX (1:1000, Merck, cat# 05-636-1) and α-Tubulin (1:1000, Merck, cat# CP06-100UG) overnight at 4°C. Membranes were washed three times with TBE and incubated with the appropriate secondary antibody conjugated with horseradish peroxidase (anti-rabbit, cat# 1706515; anti-mouse, cat# 1706516; Bio-Rad Laboratories). The SuperSignal West Pico kit (Thermo Fisher Scientific) was used for immunodetection in a ChemiDoc Touch imaging system (Bio-Rad Laboratories). Band density was quantified in image Lab 6.0.1 software (Bio-Rad Laboratories). In tissue, MCM6 and γH2AX band intensities were normalized to α-tubulin. In cell culture experiments (ACH-3P cells), MCM6 and γH2AX band intensities were normalized to Ponceau S to avoid insulin and etoposide effects on α-tubulin [21,22]. To account for inter-membrane variation, data were normalized to an internal standard (placenta tissue or untreated ACH-3P protein lysate cultured at 6.5% oxygen). For graphical representation, results were expressed relative to vehicle control at 24 h (insulin experiment) or 2.5% oxygen (oxygen exposure experiment).

### Immunostaining

Paraffin embedded placental tissues were cut into 3.5 µm sections and placed on Superfrost Plus slides (Menzel, Braunschweig, DE). Heat induced antigen-retrieval was performed in citrate buffer (pH 6.5) in a decloaking chamber (Biocare Medical, Concord, CA, U.S.A.) for 15 min at 120°C. Peroxidase and protein blocking solutions (Thermo Fisher Scientific) were added prior to antibody incubation. For immunohistochemistry, tissue sections from two different placentas were incubated with primary antibodies (MCM6 1:500, Abcam, cat# ab280215; MCM3 1:500, Abcam, cat# ab40125) for 1 h at room temperature followed by 15 min incubation with HRP polymer (Dako, Glostrup, Denmark) and 5 mi AEC single solution (Thermo Fisher Scientific). Nuclei were counterstained with Mayer’s haematoxylin (Gratt Koller, Absam, Austria). Images were acquired using a 40× objective in a Zeiss Axiophot microscope (Zeiss, Oberkochen, Germany) equipped with a digital camera (Olympus, Tokyo, Japan) using the AxioVision software (Zeiss, Oberkochen, Germany).

For immunofluorescence, tissue section from 4 different first trimester placentas were incubated with primary antibodies (MCM6 1:500, Abcam, cat# ab201683; γH2AX, 1:100, Merck, cat# 05-636-1) overnight at 4°C and incubated with secondary antibody (1:1000 anti-rabbit Alexa Fluor 568, cat# 11011; 1:200 anti-mouse Alexa Fluor 488, cat# 11001, Thermo Fisher Scientific) for 1.5 h. Nuclei were stained with 4′,6′-diamidino-2-phenylindol (DAPI). To reduce auto-fluorescence, the tissue was incubated for 5 min in 0.3% Sudan Black B (Sigma-Aldrich) dissolved in 70% (v/v) ethanol. For overall MCM6 and γH2AX, images were acquired using a 40× objective in a Zeiss Axiophot microscope (Zeiss) equipped with a digital camera (Olympus) using AxioVision software (Zeiss). For visualization purposes, the brightness/contrast was adjusted *a posteriori* using the OlyVia software (Olympus, Tokyo, Japan). For co-localization analysis, images were acquired with A1R confocal microscope (Nikon CEE GmbH, Vienna, Austria) using a 100× objective and same set ups for all images. Three images of different regions were taken for each sample. Co-localization was assessed with ImageJ Fiji software [23]. A scan line was drawn in the area of interest and the intensity (a.u.) per pixel (µm) quantified. The results were plotted in GraphPad Prism (Version 8.4.0, San Diego, CA, U.S.A.). Z-stacks were used to build 3D images with NIS-Elements software (Nikon). For visualization purposes, the brightness/contrast was adjusted *a posteriori* in ImageJ Fiji, also stated in figure legends as applies.

To investigate γH2AX and MCM6 co-localization in response to different DNA damage inducers, ACH-3P cells were incubated with primary antibodies (MCM6 1:100, Abcam, cat# ab201683; γH2AX, 1:100, Merck, cat# 05-636-1) for 1 h at room temperature followed by secondary antibody incubation (1:200 anti-rabbit Alexa Fluor 568, cat# 11001; 1:200 anti-mouse Alexa Fluor 633, cat# A-21050; Thermo Fisher Scientific) for 1.5 h. Images were acquired with A1R confocal microscope and processed as above described.

### Statistical analysis

Normal distribution of the variables and residuals was assessed by Shapiro–Wilk test and Q-Q plot examination. Mann–Whitney *U* test was used to compare two groups and one-way ANOVA (Bonferroni multiple comparison test) or Kruskall–Wallis test (Dunn’s multiple comparison test) to compare more than two groups. Correlations were tested using Pearson’s or Spearman’s correlation as appropriate. A *P*<0.05 was considered significant. Statistical analysis was performed in SPSS (Version 28.0.1.0) and GrapPhad Prism (Version 8.4.0).

## Results

### Study participants

We performed untargeted proteomics analysis on 19 first trimester placentas. Clinical traits including BMI, C-peptide and IS_HOMA_ of the women undergoing elective terminations are described in Table 1.

### The placental proteome differs between women with low and high insulin sensitivity

**Figure 1:**
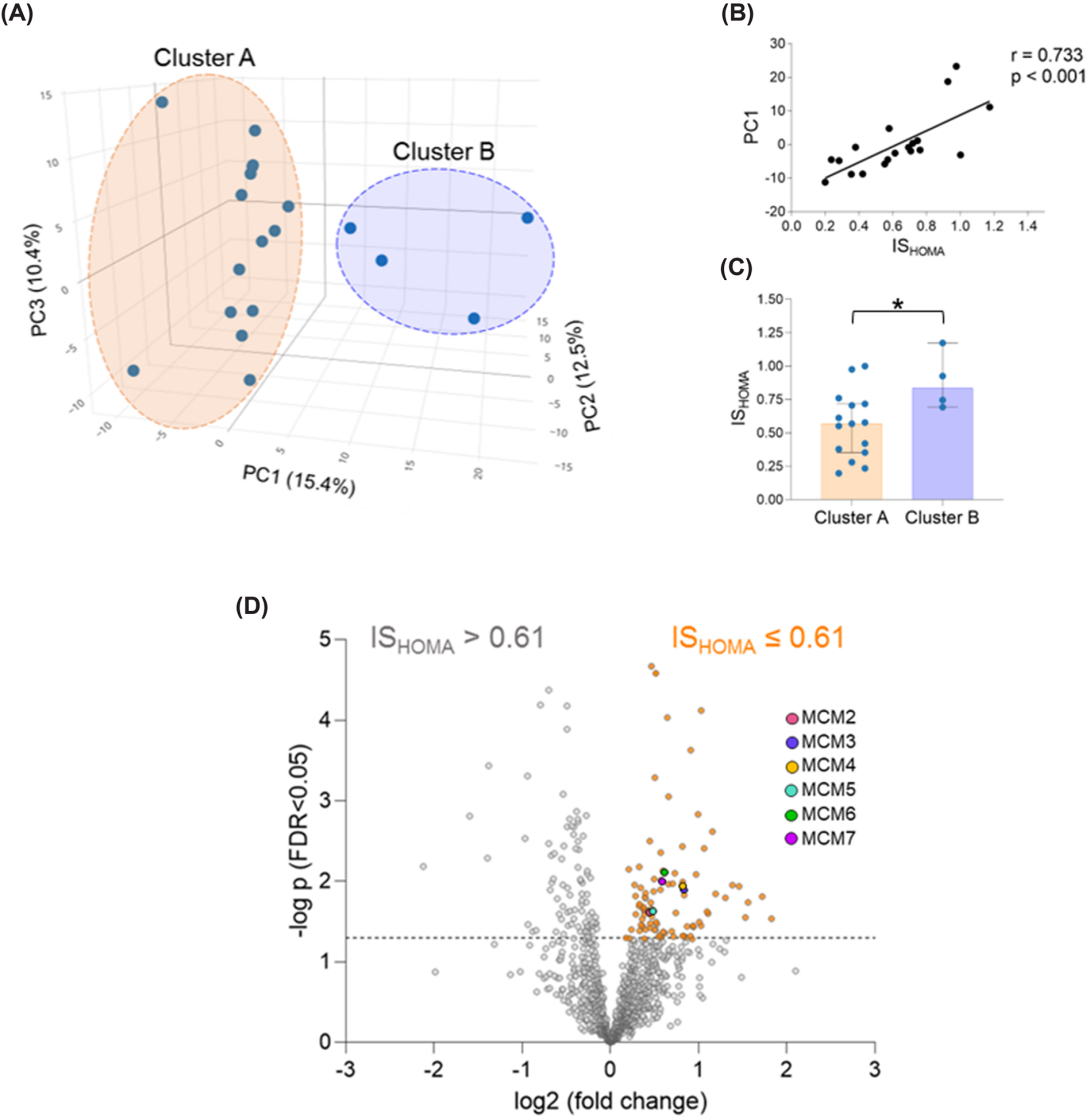
Maternal insulin sensitivity associates with changes in the placental proteome The proteome of first trimester placental tissue (*n*=19) was analyzed by untargeted proteomics (MS/MS). (**A**) Principal Component Analysis demonstrated two clusters. (**B**) IS_HOMA_ was the clinical trait that better correlated with PC1 (**C**) Maternal IS_HOMA_ was the only parameter significantly (*P*<0.05) different between clusters (Mann–Whitney *U* test). Individual data as well as median and 95% confidence intervals are presented. (**D**) The samples were classified into high (IS_HOMA_≥0.61) or low (IS_HOMA_<0.61) insulin sensitivity based on the median IS_HOMA_ value (median IS_HOMA_ = 0.61) and their proteome compared. Proteins above the dotted line are significantly (*P*<0.05) enriched. Proteins significantly enriched in the low IS_HOMA_ group are colored in orange. BMI, body mass index; IS_HOMA_, homeostatic model assessment of insulin; ns, non-significant; *P<0.05.

The proteome of 19 first trimester placentas was analysed by untargeted proteomics. Principal component analysis (PCA) demonstrated two clusters of placental samples (Cluster A: *n*=15; Cluster B: *n*=4) (Figure 1A). To identify the driver of cluster separation in PC1, clinical traits of the women (BMI, leptin, glucose, C-peptide and IS_HOMA_ index) and gestational age were correlated with PC1 coordinates. Insulin sensitivity, i.e., IS_HOMA_, was the clinical trait that best correlated (highest correlation coefficient) with PC1 (*r* = 0.733, *P*<0.001) (Figure 1B). When the clinical traits were compared between clusters using a group-comparison test, only IS_HOMA_ was significantly different (*P*=0.049) between clusters. IS_HOMA_ was higher in women whose placentas grouped in cluster B (Figure 1C). BMI did not significantly differ between clusters despite a correlation between BMI and IS_HOMA_ (*r* = −0.556, *P*=0.013).

Thereafter, we used the median IS_HOMA_ of the cohort (median IS_HOMA_ = 0.61) as cut-off to classify placental samples of women into those with IS_HOMA_>0.61 or IS_HOMA_≤0.61 and denoted them as ‘high’ and ‘low’ insulin sensitivity, respectively. Clinical traits of women in these two groups are shown in Table 2. Differential expression analysis compared the proteomes between the two groups. It identified 86 proteins enriched in the group of placentas of women with low insulin sensitivity as represented in a volcano plot (Figure 1D).

**Table 2:** Clinical traits of the women whose placental tissue was used for proteomics stratified by IS_HOMA_

	Discovery cohort (*n*=19)		
	IS_HOMA_ > 0.61 (*n*=9)	IS_HOMA_ ≤ 0.61 (*n*=10)	
	Median; IQR	Median; IQR	*P*-value
Gestational age (days)	35 (35–43)	45 (35–48)	0.113
Maternal age (years)	35 (25–39)	32 (29–35)	0.780
BMI (kg/m^2^)	21.2 (19.7–23.2)	28.7 (23.3–32.4)	0.079
Leptin (ng/ml)	9.3 (2.7–14.2)	19.2 (14.0–28.5)	0.055
Glucose (mmol/l)	5.2 (4.1–5.8)	5.5 (4.8–6.4)	0.278
C-peptide (pmol/l)	308.5 (277.6–413.0)	599.2 (510.7–760.8)	**<0.001**
IS_HOMA_ index	0.75 (0.7–0.98)	0.34 (0.26–0.57)	**<0.001**

Maternal clinical traits (median, IQR) of the women whose placental tissue was subjected to untargeted proteomics analysis (*n*=19) stratified by IS_HOMA_.

BMI, body mass index; IS_HOMA_, homeostatic model assessment of insulin sensitivity; IQR, interquartile range; *P*: *P*-value.

### DNA replication and cell cycle pathways are up-regulated in the placental proteome of women with low insulin sensitivity

**Figure 2:**
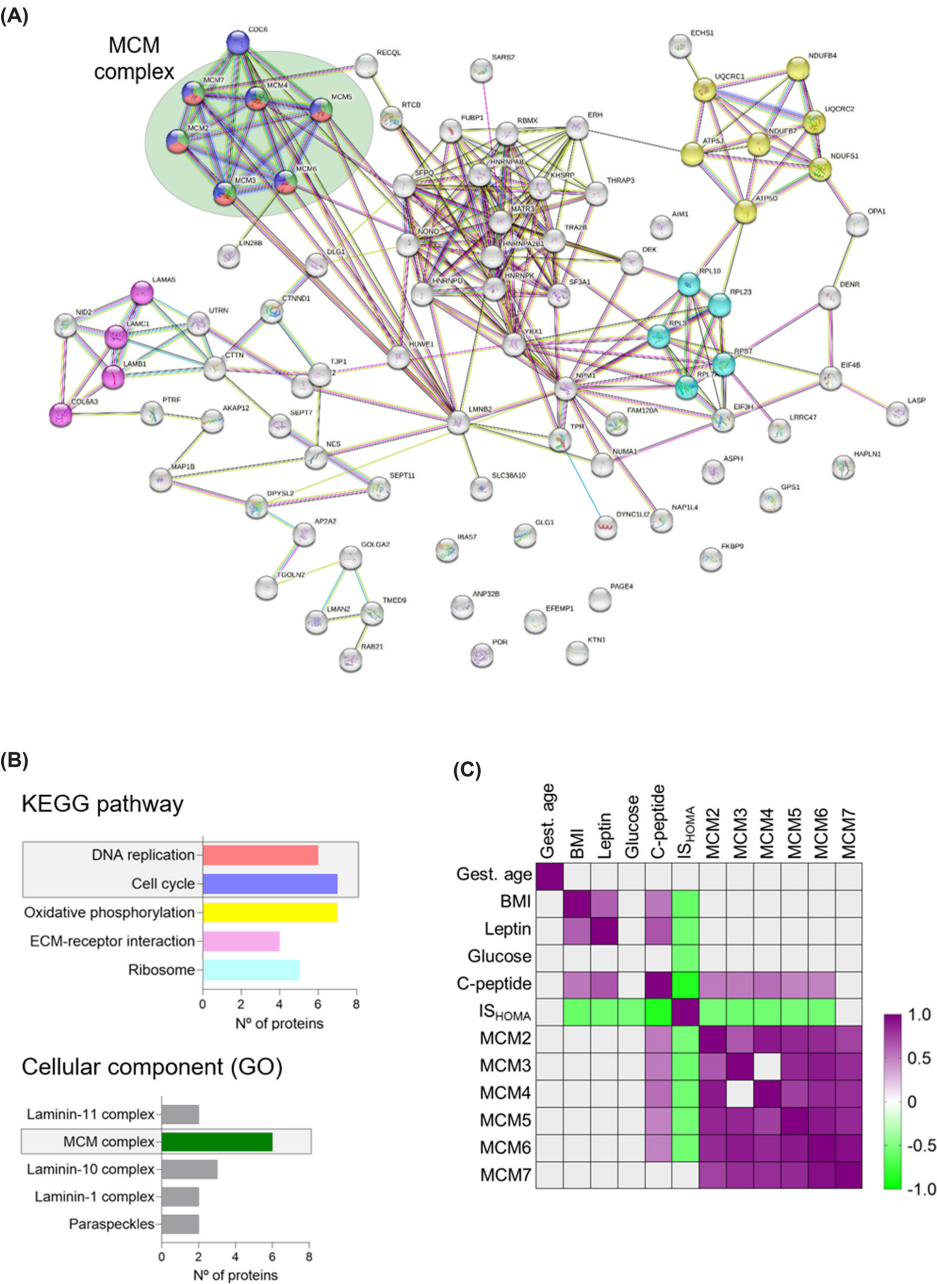
DNA replication and cell cycle pathways are up-regulated in the placental proteome of women with low insulin sensitivity and the MCM-complex drives this enrichment (**A**) Proteins enriched (*n*=86, *P*<0.05) in the low IS_HOMA_ group (IS_HOMA_<0.61) were selected for further network analysis (String V.11.5). (**B**) DNA replication, cell cycle and ribosome pathways were significantly enriched KEGG pathways in the low IS_HOMA_ group. Six proteins from the MCM-complex (MCM2-7) were the drivers for enrichment of DNA replication and cell cycle pathways (Gene Ontology). (**C**) Clinical traits known to be dysregulated in maternal obesity in the first trimester of pregnancy were correlated with raw LFQ values (Label Free Quantification; semi-quantitative measure of concentration) of MCM2-7 proteins. All proteins of the MCM-complex, except MCM7, were positively correlated with C-peptide and inversely correlated with IS_HOMA_. Most individual MCM proteins were positively correlated with each other. ECM, Extracellular matrix; GO, Gene Ontology; IS_HOMA_, homeostatic model assessment of insulin; KEGG, Kyoto Encyclopedia of Genes and Genomes.

Using STRING network analysis [18], we explored associations between the 86 proteins enriched in the group of placentas of women with low insulin sensitivity. The individual proteins up-regulated in the low ISHOMA group and their interrelationship are visualized in Figure 2A. Using the Kyoto Encyclopedia of Genes and Genomes (KEGG) we found ‘DNA replication’, ‘cell cycle’ and ‘ribosome processes pathways’ significantly enriched in placentas of women with low ISHOMA (Figure 2B). Terms related to DNA replication, DNA damage and DNA repair were recurrent in other databases such as gene ontology and reactome pathways (Supplementary Table S1). Proteins driving DNA-related pathways were MCM2, MCM3, MCM4, MCM5, MCM6 and MCM7. The MCM proteins constitute the MCM-complex (Supplementary Figure S2) and are components of the pre-replicative complex at the origins of replication during the G1 phase of the cell cycle [24]. MCM proteins are also known to participate in DNA damage repair [25–27].

Next, we sought to test for correlations between maternal clinical traits and MCM2-7 protein levels. IS_HOMA_ was inversely correlated with MCM2-6 suggesting that placentas of women with low insulin sensitivity have increased MCM2-6 protein levels. C-peptide, a component of IS_HOMA_ calculation, was positively correlated (Figure 2C and Supplementary Table S2). MCM7 was not significantly correlated with any clinical trait. Moreover, protein levels of each MCM correlated with those of all other MCMs tested except of MCM3, which did not correlate with MCM4 (Supplementary Table S3). This suggests that MCM2-7 act synergistically as part of the MCM2-7 heterohexamer (MCM-complex) [24,28]. Because of this, we selected MCM6, the MCM most enriched in the low IS_HOMA_ group (proteomic analysis), as a representative of the MCM-complex for further investigation (Supplementary Figure S2).

### γH2AX and MCM6 co-localize in human first trimester cytotrophoblast cells

**Figure 3:**
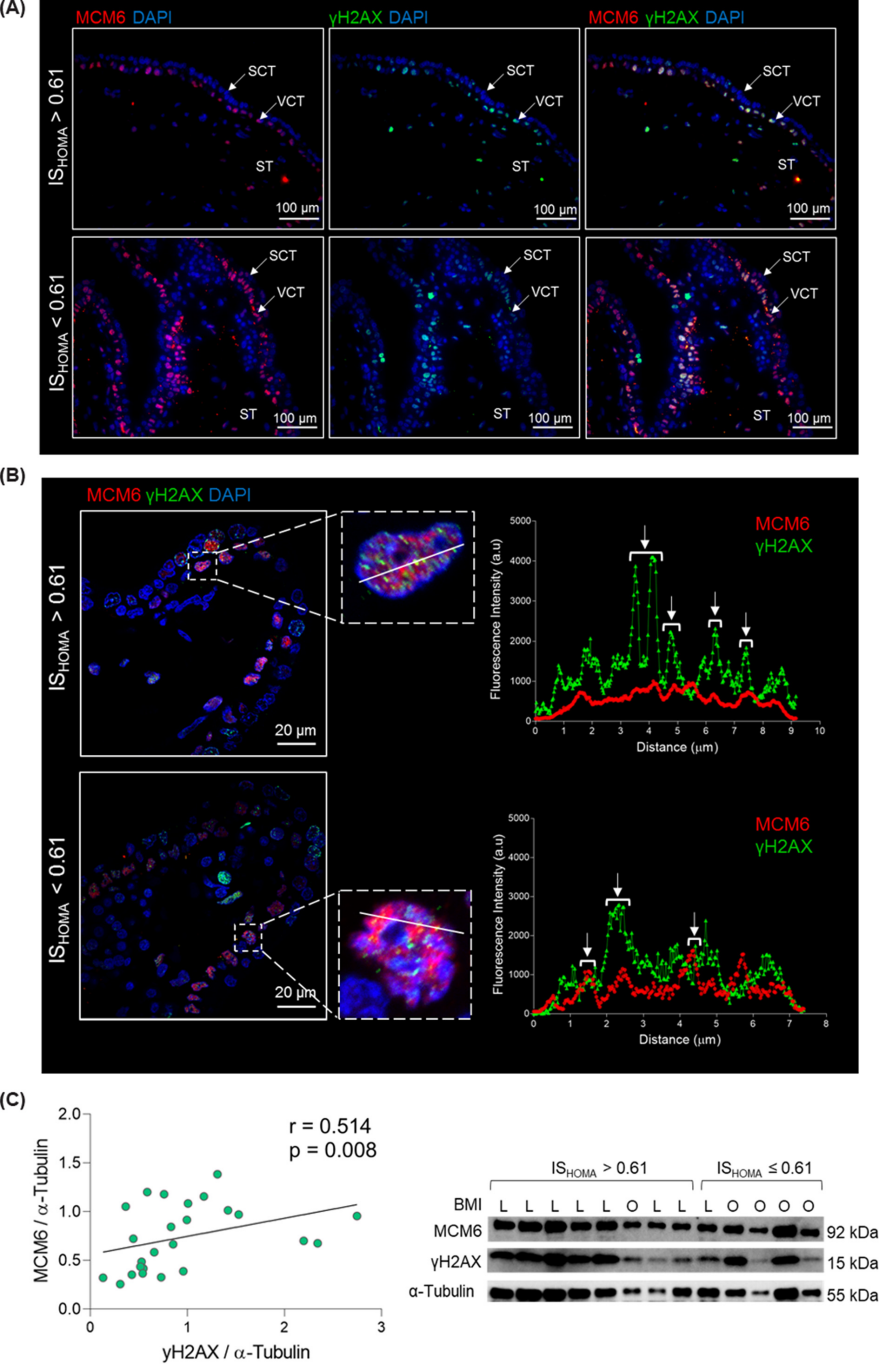
γH2AX and MCM6 co-localize to the nuclei of villous cytotrophoblast cells in the first trimester placenta Placenta tissue of high (*n*=2) and low (*n*=2) insulin sensitive pregnant women was stained with anti-MCM6, anti-γH2AX antibodies and DAPI (nucleus). Representative images are shown. (**A**) Tissue overview of MCM6 and γH2AX staining acquired in a light microscope (40×). MCM6 and γH2AX localize predominantly to the nuclei of cytotrophoblast cells. Some MCM6 foci are visible in the cytoplasm (see supplementary Figure S3). (**B**) For co-localization assessment, z-stacks were acquired in a confocal microscope (100×). The line scan tool (ImageJ, FIJI) was used to measure fluorescence intensity (a.u.) per pixel (µm) and the data plotted using GraphPad prism. (**C**) First trimester placental lysates (*n*=23) were resolved in SDS-PAGE and probed with anti-MCM6, anti-γH2AX and anti-α-tubulin antibodies. Band intensity was quantified and normalized to α-tubulin. MCM6 and γH2AX protein levels were correlated (Spearman’s correlation). Brightness and contrast adjusted for visualization purpose. IS_HOMA_, homeostatic model assessment of insulin; L, lean; O, overweight-obese.

Because the MCM-complex has a role in the repair of DNA double strand breaks, we hypothesized that increased MCM levels in placentas of women with low insulin sensitivity reflect increased DNA damage in these placentas. To fulfil its function, MCM6 has to be present in close proximity to DNA damage sites (γH2AX foci) [29,30]. Therefore, we ascertained the cellular and subcellular localization of MCM6 and γH2AX in tissue sections from four different first trimester placentas (Figure 3A; Supplementary Table S5 and Supplementary Figure S2). The MCM6 protein was mainly localized to the nuclei of villous cytotrophoblast cells, the proliferative cell type of the placenta. Some nuclei of stromal cells were also immunopositive for MCM6. Surprisingly, some MCM6 foci were also detected in cytoplasmic regions (Supplementary Figure S3). Similarly, γH2AX foci were also predominantly found in the nuclei of villous cytotrophoblast cells with some nuclei in the syncytiotrophoblast and stroma also immunopositive for γH2AX.

We performed co-localization analyses to investigate whether MCM6 and γH2AX are in close proximity (Figure 3B). We found that MCM6 and γH2AX fluorescence intensities overlap in villous cytotrophoblast nuclei, indicating co-localization of MCM6 and γH2AX in this specific cell type. This co-localization was found in placentas of women with low and high IS_HOMA_ (Figure 3B).

Furthermore, γH2AX significantly correlated with MCM6 protein levels in total placental tissue (*n*=23; *r* = 0.514; *P*<0.01), suggesting that increased DNA damage associates with increased MCM6 protein levels (Figure 3C and Supplementary Table S4).

### DNA damage induction in ACH-3P cells

**Figure 4:**
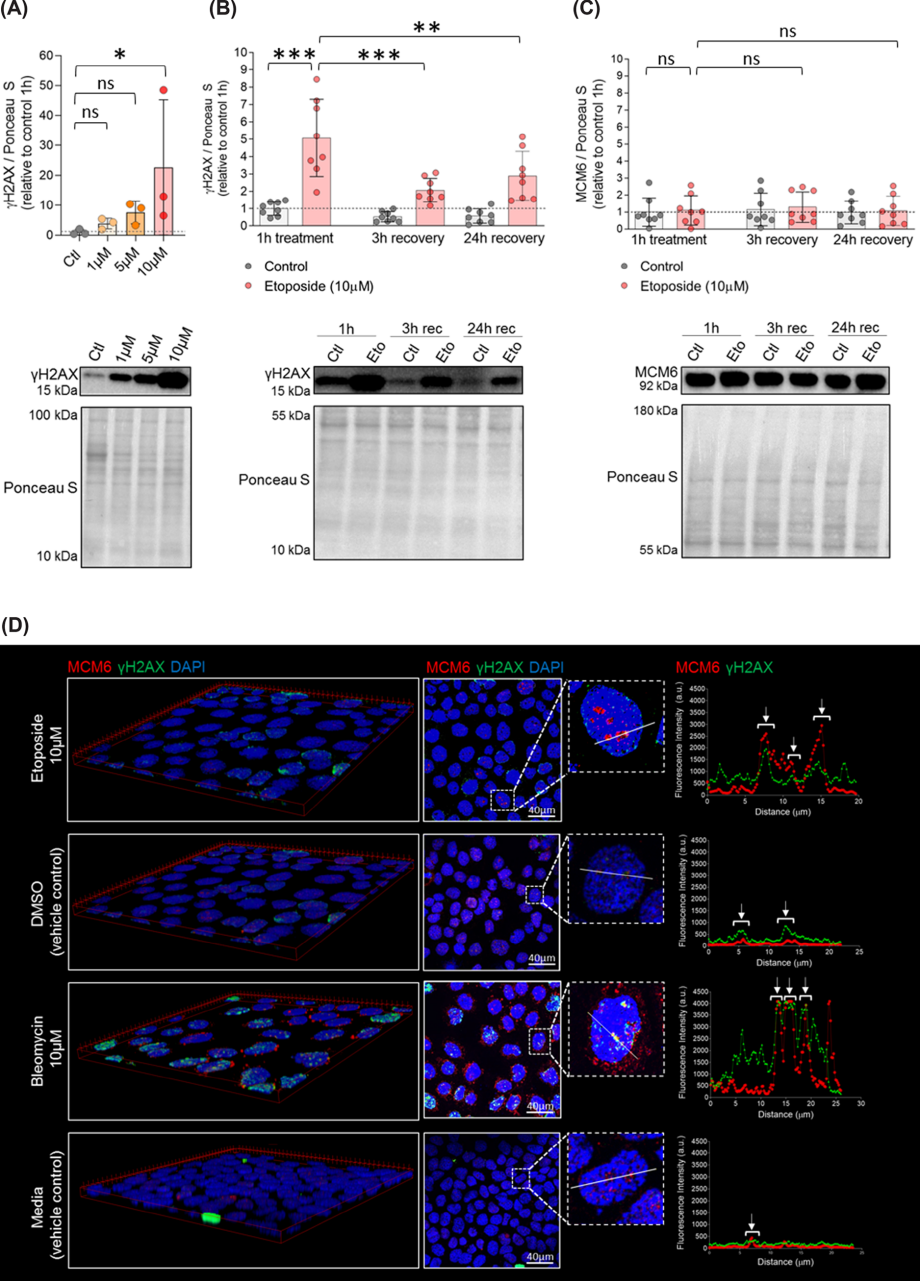
Etoposide induces DNA damage in ACH-3P cells and the damage is repaired within 3 h (**A**) ACH-3P cells (*n*=1 experiment; in triplicates) were cultured at 6.5% O_2_ in absence (control) or presence of etoposide (1, 5 and 10 µM) for 1 h. Cells were harvested immediately and cell lysates subjected to Western blotting. DNA damage (measured as γH2AX) was quantified and protein levels normalized to Ponceau S. Data are presented as mean ± SD. (**B,C**) After 1 h of treatment (control or 10 µM etoposide), cells (*n* = 3 experiments; in triplicates) were allowed to recover in fresh media for 3 or 24 h. Thereafter, cells were harvested and cell lysates subjected to Western blotting using anti-γH2AX and anti-MCM6 antibodies. Results are shown relative to control at 1 h. Data are presented as mean ± SD. One-way ANOVA with post hoc test and Bonferroni correction was used to compare protein levels between control after 1 h of exposure (reference) and etoposide after 1 h of exposure and 3 and 24 h of recovery. (**D**) MCM6 is recruited to sites of DNA damage. ACH-3P cells (*n* = 1 experiment, in triplicates) were cultured at 6.5% O_2_ in presence of etoposide (10 µM), bleomycin (10 µM) or vehicle control (DMSO and F12 media, respectively) for 1 h. Thereafter, cells were allowed to recover in fresh media (F12 media) for 1 h. Subsequently, cells were incubated overnight with anti-MCM6 and anti-γH2AX. DAPI was used to visualize nuclei. Images were acquired with a confocal microscope. 3D images and co-localization analysis in ImageJ - Fiji. Arrows indicate regions, i.e., pixels, where γH2AX and MCM6 signals co-localize. Brightness and contrast of immunofluorescence and Ponceau S staining adjusted for better visualization. *P<0.05, **P<0.01, ***P<0.001. ns: non-significant. Ctl: vehicle control (DMSO); Eto: etoposide; Rec: recovery.

To study the dynamics of DNA damage and DNA damage repair in the first trimester placenta, the first trimester trophoblast cell line ACH-3P was used [31]. In a positive control experiment, exposure to etoposide, a topoisomerase II inhibitor, for 1 h was sufficient to induce DNA damage (γH2AX increase) (Figure 4A). DNA damage was partially repaired after 3 h of culture in absence of this drug (Figure 4B). However, this was not accompanied by changes in MCM6 levels (Figure 4C).

To address whether MCM6 can be actively recruited to sites of DNA damage, ACH-3P cells were challenged with etoposide (causes DNA damage at origin of replication sites) or bleomycin (causes damage at random sites of the chromosomes). Both treatments induced formation of γH2AX foci and γH2AX and MCM6 co-localized in both experimental conditions. Visual inspection of co-localization analysis after bleomycin treatment indicates that MCM6 can be recruited to sites of DNA damage (Figure 4D).

### Insulin and oxygen do not induce DNA damage in ACH-3P cells

**Figure 5:**
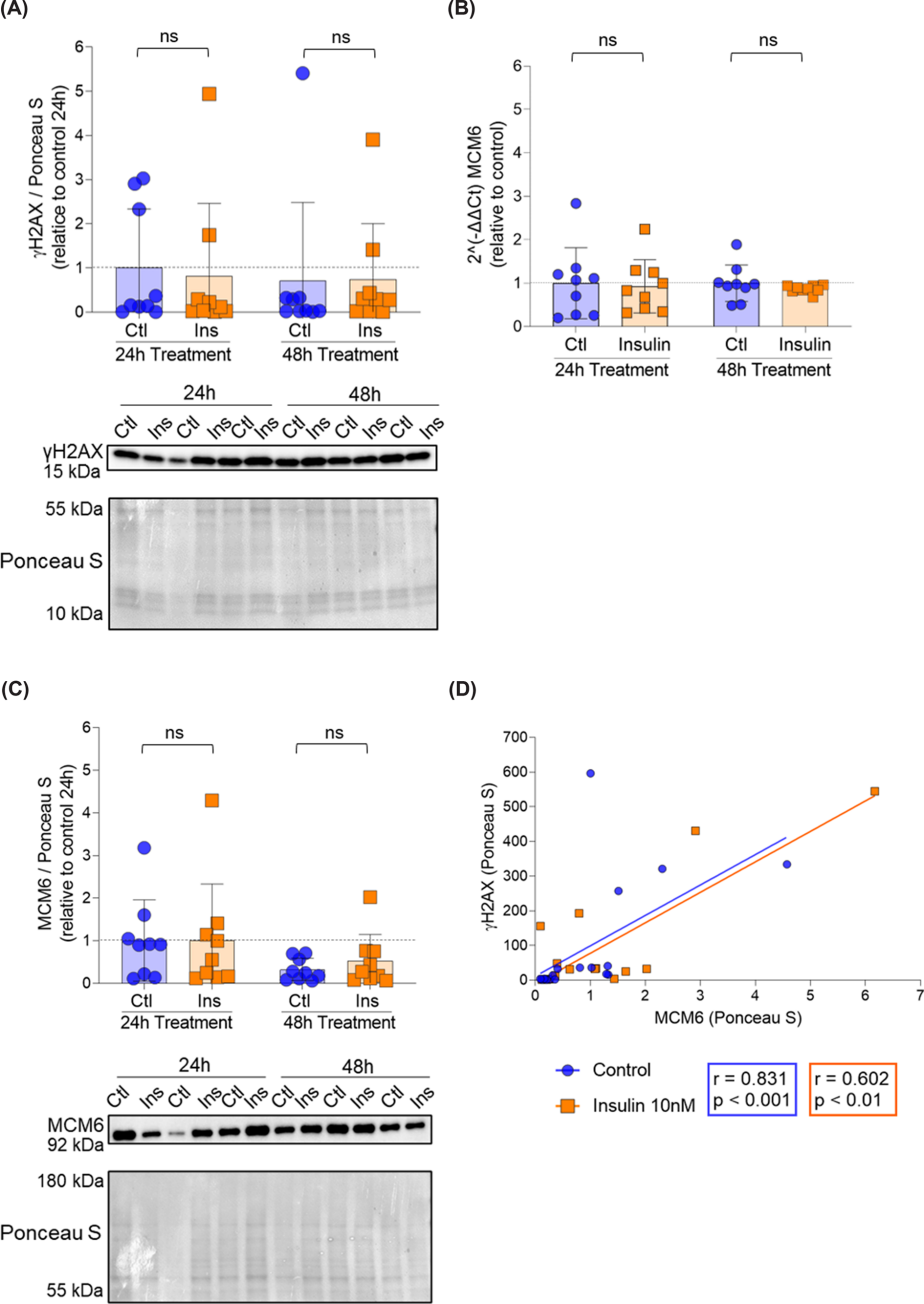
The correlation between γH2AX and MCM6 is independent of insulin ACH-3P cells (*n* = 3 experiments; in triplicates) were cultured at 6.5% O_2_ for 48 h to accommodate them to the oxygen tension. After 72 h, cells were treated with vehicle control (F12 media) or 10 nM insulin. Cells were harvested after additional 24 and 48 h and cell lysates subjected to RT-qPCR and western blotting. (**A**) DNA damage (measured as γH2AX) was quantified by Western blotting and protein concentration normalized to Ponceau S. (**B**) MCM6 expression was quantified by RT-qPCR and normalized to housekeeping genes peptidyl-prolyl isomerase (PPIA) and TATA-binding protein (TBP). (**C**) MCM6 was quantified by western blotting and protein concentration normalized to Ponceau S. (**D**) γH2AX and MCM6 protein levels were correlated using Spearman correlation. Results (A–C) are shown relative to control 24 h. Data are presented as mean ± SD. Kruskal–Wallis test was used to compare expression and protein levels between controls and insulin treated samples. Brightness and contrast of Ponceau S staining adjusted for better visualization. ns: non-significant.

Insulin sensitivity (IS_HOMA_ index) is calculated using glucose and C-peptide values. Because insulin sensitivity itself cannot induce changes in the placental proteome, we speculated that the changes in MCMs protein levels must be mediated by glucose or C-peptide. Since only C-peptide (surrogate of insulin) was significantly correlated with MCMs protein levels (Figure 2), we chose insulin to challenge ACH-3P cells and obtain mechanistic insight. Insulin exposure for 24 and 48 h did not cause DNA damage (Figure 5A) and it did not affect MCM6 mRNA expression (Figure 5B) nor protein levels (Figure 5C). There was a significant correlation between γH2AX and MCM6 protein levels, but it was independent of insulin treatment (Figure 5D).

**Figure 6:**
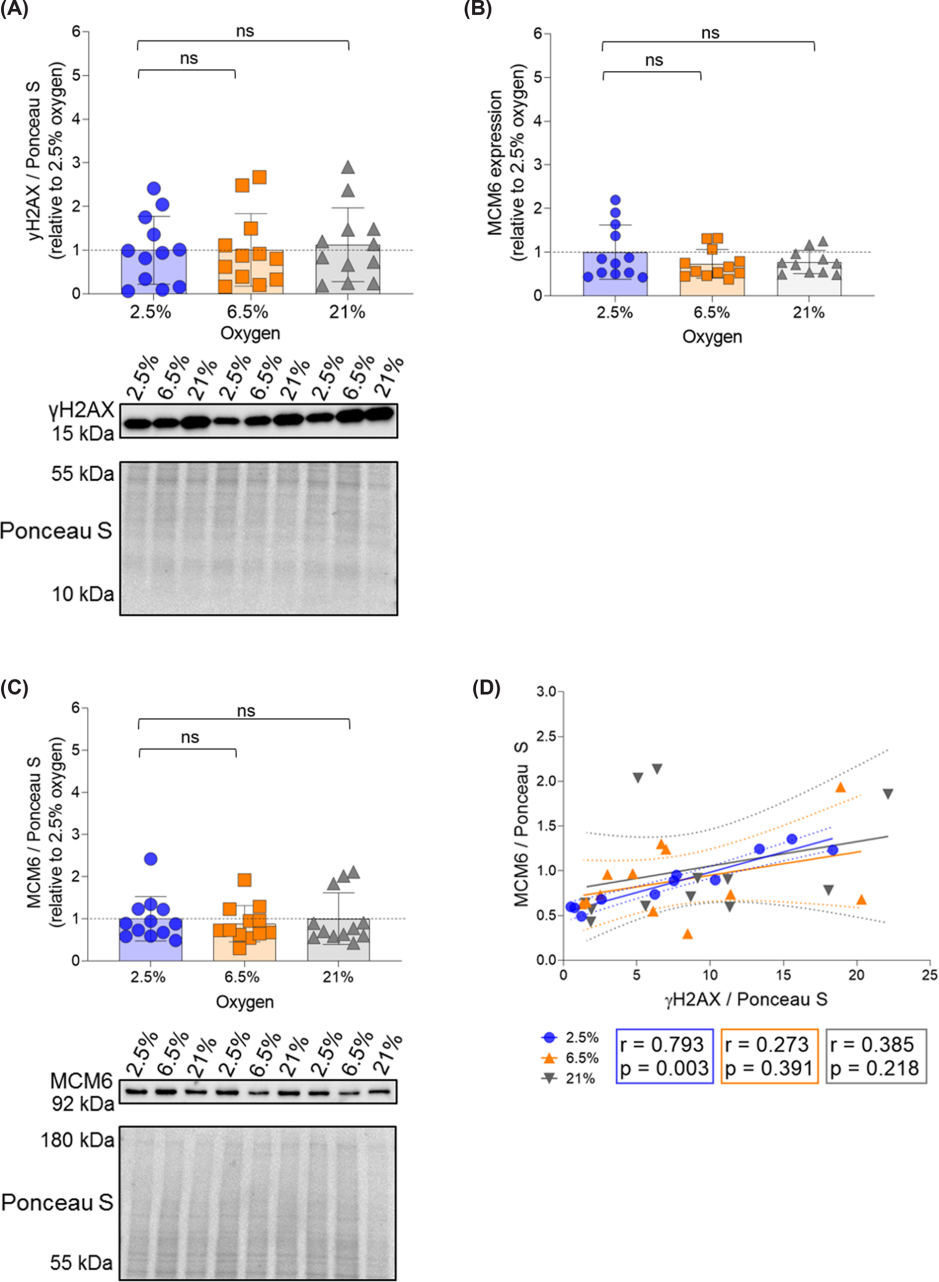
The correlation between γH2AX and MCM6 is independent of oxygen tension ACH-3P cells (*n* = 4 experiments; in triplicates) were cultured at 2.5% O_2_ for 48 h. Afterward, one plate per experiment was kept at 2.5%, while the other plates were transferred to 6.5% or 21% for 48 additional hours. Thereafter, cells were harvested and cell lysates subjected to PCR or Western blotting. (**A**) DNA damage (measured as γH2AX) was quantified and protein level normalized to Ponceau S. (**B**) MCM6 expression was quantified by RT-qPCR and normalized to the house keeping genes PPIA and TBP. (**C**) MCM6 was quantified and protein level normalized to Ponceau S. (**D**) γH2AX and MCM6 protein levels were correlated using Spearman correlation. Results are shown relative to 2.5% O_2_. Data are presented as mean ± SD. Kruskal–Wallis test was used to compare protein levels between samples at 2.5% O_2_ (reference category) and samples exposed to 6.5% and 21% O_2_. Brightness and contrast of Ponceau S staining adjusted for better visualization. ns: non-significant.

Evidence suggests a delay in spiral artery remodelling of placentas exposed to maternal obesity [32]. Consequently, these placentas are exposed to lower oxygen tension for a prolonged time. We speculated that the prolonged exposure to low oxygen combined with hypoxia-reoxygenation [33] induces increased MCMs expression and protein levels. Culturing ACH-3P cells at 2.5% O_2_, i.e. physiological oxygen tension before spiral artery remodeling, during 96 h did not result in differences in γH2AX protein levels (Figure 6A), MCM6 mRNA expression (Figure 6B) or protein levels (Figure 6C) compared with cells transferred to 6.5% (physiological oxygen tension after the spiral artery remodelling) or 21% (hyperoxygenation) for 48 h. Again, γH2AX and MCM6 protein levels were significantly correlated, but only at 2.5% oxygen (Figure 6D). However, large variation of the data may have precluded correlation at 6.5% and 21% oxygen.

## Discussion

This is the first untargeted study analysing the proteome of human first trimester placentas exposed to an obesogenic environment *in utero*. We found that exposure to low maternal insulin sensitivity associates with changes in the placental proteome. These changes include up-regulation of cell cycle and DNA damage repair pathways. We identified the MCM-complex as a key player in these pathways. While the MCM-complex has been studied intensively in cancer, where it is overexpressed and used as diagnostic marker of disease progression [34], its potential role in metabolic pathophysiology has not been described yet.

The MCM-complex is formed by six homologous proteins that are recruited to the origins of replication to initiate DNA replication in the S-phase of the cell cycle [35]. However, MCM proteins are estimated to be 40- to 100-fold more abundant than the number of origins of replication in the cell and only a fraction of MCMs is associated with chromatin during the S-phase [27,36]. This phenomenon is known as the MCM-paradox and suggests alternative roles for MCM proteins. These alternative roles include maintaining chromosome stability and participating in DNA damage repair by interacting with checkpoint regulators (e.g., radiation (Rad)17) [37] and proteins involved in DNA damage repair (ATM, ataxia telangiectasia mutated; ATR, ataxia telangiectasia and rad3-related protein; TP53, tumor protein 53; 53BP1, tumor protein 53 binding protein) [38–40]. Of note, protein levels of Rad52, which together with Rad 51 is critical for homologous recombination, are highly elevated in placentas of women with obesity [11]. Our finding of a correlation between γH2AX (marker of DNA damage) and MCM6 protein level in the first trimester placental tissue together with the co-localization of both proteins in villous cytotrophoblast nuclei suggests that MCMs participate in repairing DNA damage in this cell type. However, this notion requires formal testing.

The gene coding for MCM6 is located at chromosome 2, in proximity to the lactase gene (*LCT*). Because *LCT* has been shown to be overexpressed in people with obesity [41,42], the MCM6 increase found here may be genetically driven. However, each MCM gene is located on a different chromosome, excluding the hypothesis of MCM6 overexpression only due to genetic background. Moreover, the correlation between protein levels of individual MCMs in the first trimester placenta argues for a synergistic action of MCMs as part of the MCM-complex, rather than they executing individualized functions. A further argument for a synergistic activity of MCM2-7 is that MCM proteins, which are not part of the MCM-complex but rather act individually (MCM1, MCM8 and MCM9) [24], were not enriched in placentas of women with low insulin sensitivity.

To investigate whether MCMs role in repairing DNA damage is limited to breaks occurring at origins of replication or whether MCMs can be actively recruited to sites of DNA damage, we challenged ACH-3P cells with etoposide (topoisomerase II inhibitor) and bleomycin (cleaves DNA in the presence of iron and oxygen). Co-localization of γH2AX and MCM6 in cells treated with bleomycin shows that MCMs can be recruited to sites of DNA damage which are not located at origins of replication. This indicates that MCMs participate in DNA damage repair also at other locations in the chromatin. Interestingly, MCM6 foci also localized to the cytosol and this was especially evident in cells treated with bleomycin. Re-examining stained tissue sections for MCM6, we corroborated that cytoplasmic localization also occurs in placental tissue. The role of cytoplasmic MCM6 foci is unclear, but increasing evidence suggests a role in the stability of stress granules [42]. Further studies in this direction are required and may establish a new role for MCM proteins as coordinators of stress response pathways and DNA replication.

The IS_HOMA_ index is calculated from fasting glucose and C-peptide values. Because C-peptide, the by-product of insulin synthesis, but not glucose, was significantly correlated with MCMs protein levels, we speculated that increased MCMs levels in placentas of women with low insulin sensitivity might be the result of hyperinsulinemia rather than hyperglycemia. To establish a causal link between hyperinsulinemia and MCMs up-regulation, we challenged the ACH-3P cell line with insulin. However, we did not detect an increase in DNA damage nor MCM6 concentration under these experimental conditions, although ACH-3P are insulin responsive [43,44].

MCM expression is up-regulated in proliferating cells [45,46]. Cytotrophoblast cells have been shown to proliferate under low oxygen tension (2%) and to differentiate when oxygen tension rises (20%) [47]. We speculated that the increased MCM levels in placentas of women with low insulin sensitivity might reflect low oxygenation in these placentas. This could be caused by delayed opening of the spiral arteries, which has been suggested in pregnancies complicated by obesity [32]. However, in ACH-3P cells, a suitable cell model for first trimester proliferating cytotrophoblast cells [31] that respond to insulin and oxygen tension changes [43,44], exposure to low oxygen tension (2.5%) did not increase DNA damage nor MCM6 concentration, despite a correlation between γH2AX and MCM6 only at this specific oxygen tension. The MCM paradox as well as the short exposure time *in vitro* mimicking acute effects of the environment may account for the lack of change in MCM6 concentration in ACH-3P cells under the tested experimental conditions. Indeed, MCM6 expression in a mouse model of obesity-insulin resistance (BTBR ob/ob) is increased in the pancreatic islets and adipose tissue at 10 weeks, but not at 4 weeks of age [48]. Similarly, in humans with overweight-obesity, hepatic MCM2 and muscular MCM4 are overexpressed in the low insulin sensitive compared with the normal insulin sensitive group [49].

This is the first study on how the maternal environment often associated with obesity alters the placental proteome and function already in the first trimester. An important strength is that we considered maternal clinical traits like leptin, glucose, C-peptide and insulin sensitivity and not only BMI. This is important because individuals with high BMI can still be metabolically healthy [50], making BMI a poor metabolic health indicator [51].

We included samples of a narrow gestational age range (5^+0^–6^+6^ weeks) to prevent confounding effects of a wider gestational age range. This is important to avoid differences caused by the transition from histiotrophic to haematotrophic nutrition and rise in oxygen tension in the intervillous space between weeks 6 and 12 of pregnancy [52,53]. Such transitions would hide the association between placenta proteins and a specific maternal phenotype [54]. Rather, we captured effects predominantly caused by exposure to the maternal obesogenic environment *in utero*. Similar to our study, a gene expression microarray found cell cycle and DNA metabolism pathways up-regulated in first trimester (6^+3^–8^+3^ days) compared with second trimester (15^+4^–16^+3^ weeks) placentas [55], indicating that up-regulation of these pathways is common in the first trimester period. Unfortunately, that study did not provide information on maternal characteristics, which precludes interpreting whether their findings are due to an overrepresentation of women with obesity in their cohort.

A limitation of our study is the relatively small sample size, which precluded analysing potential fetal sex effects on the placenta proteome. However, MCMs expression was not significantly different in transcriptomic analysis of male and female placentas [56,57]. The small amount of tissue in this very early period also made it impossible to run all experiments on all tissue samples. It shall also be noted that immunohistochemistry was used solely for qualitative measurements.

Our *in vitro* studies failed to induce *in vitro* MCM changes in ACH-3P cells by treating them with insulin. This may be accounted for by ACH3-P cell properties and needs to be formally confirmed in another first trimester cell line model such as AL07 cells [58]. As noted above, it is important to keep in mind that these experiments mimic acute changes whereas the placentas studied here have been exposed to the obesity-associated intrauterine environment for several weeks. Hence, the *in vivo* first trimester placental findings are the result of chronic exposure. Consequently, acute *in vitro* studies using cell cultures may not be suitable to capture the homeostatic and allostatic mechanisms that are in place *in vivo* to drive placental development. Systems-wide approaches capturing these mechanisms are needed and should be the next step.

In conclusion, chronic exposure *in utero* to reduced maternal insulin sensitivity during 5^+0^– 6^+6^ weeks in the first trimester of human pregnancy induces changes in the placental proteome, especially of pathways related to DNA replication, cell cycle and DNA damage repair.

## Supplementary Material

Supplementary Figures S1-S3 and Tables S1-S5

## Data Availability

Mass spectrometry proteomics data was deposited to the ProteomeXchange Consortium via the PRIDE [16] partner repository with the data set identifier PXD056380.

## Abbreviations

53BP1: tumor protein 53 binding protein
ATM: ataxia telangiectasia mutated
ATR: ataxia telangiectasia and rad3-related protein
BRCA1: breast cancer 1
CDC25C: cell division cycle 25C
Chk1: checkpoint kinase 1
Chk2: checkpoint kinase 2
DAPI: 4′,6′-diamidino-2-phenylindol
PCA: principal component analysis
SMAD2/3: suppressor of mothers against decapentaplegic 2/3
TP53: tumor protein 53
