# Supplementary material for: MCM proteins are up-regulated in placentas of women with reduced insulin sensitivity
Source: Biosci Rep. 2024 Oct 8;44(10):BSR20240430. doi: 10.1042/BSR20240430 (PMC11461181; doi:10.1042/BSR20240430)
Supplement: Supplementary Figures S1-S3 and Tables S1-S5 [file BSR-2024-0430_supp.pdf]

Supplementary Materials

- Supplementary Table 1. Pathways and processes upregulated in placentas of women with low maternal insulin sensitivity
- Supplementary Table 2. Correlation of maternal clinical traits and MCM protein levels
- Supplementary Table 3. Correlation of MCMs protein levels
- Supplementary Table 4. Maternal clinical traits of the cohort used for western blotting
- Supplementary Table 5. Maternal clinical traits of the cohort used for immunohistochemistry
- Supplementary Figure 1. Allocation of samples to experiments
- Supplementary Figure 2. MCM3 and MCM6 localize to the nuclei of cytotrophoblast cells in the first trimester placenta
- Supplementary Figure 3. MCM6 localizes to the nuclei and cytoplasm of cytotrophoblast cells

Supplementary Table 1. Pathways and processes upregulated in placentas exposed to low maternal insulin sensitivity.

GO-term	Description	Counts in network	Strength	FDR
Biological processes (Gene Ontology)				
GO:1905663	Positive regulation of telomerase RNA reverse transcriptase activity	2 of 2	2.36	0.0145
GO:0006267	Pre-replicative complex assembly involved in nuclear cell cycle DNA replication	6 of 7	2.29	5.10e-08
GO:1902975	Mitotic dna replication initiation	3 of 4	2.23	0.00073
GO:1990428	miRNA transport	2 of 3	2.18	0.0217
GO:0000727	Double-strand break repair via break-induced replication	6 of 11	2.09	9.06e-08
GO:1902969	Mitotic dna replication	4 of 11	1.92	0.00018
GO:0006270	DNA replication initiation	6 of 32	1.63	1.10E-05
GO:0006271	DNA strand elongation involved in DNA replication	3 of 18	1.58	0.0133
GO:0048026	Positive regulation of mRNA splicing, via spliceosome	3 of 22	1.49	0.02
GO:0048025	Negative regulation of mRNA splicing, via spliceosome	3 of 23	1.47	0.0221
Molecular Function (Gene Ontology)				
GO:0003688	DNA replication origin binding	6 of 23	1.77	1.62E-06
GO:0003678	DNA helicase activity	7 of 63	1.4	9.30E-06
GO:0003697	Single-stranded DNA binding	10 of 113	1.3	2.44E-07
GO:0003729	mRNA binding	13 of 289	1.01	5.32E-07
GO:0045296	Cadherin binding	10 of 334	0.83	0.00064
GO:0005198	Structural molecule activity	16 of 635	0.76	9.07E-06
GO:0050839	Cell adhesion molecule binding	13 of 538	0.74	0.00021
GO:0003723	RNA binding	36 of 1649	0.7	2.72E-13
GO:0016887	ATPase activity	8 of 393	0.67	0.0445
GO:0017111	Nucleoside-triphosphatase activity	13 of 760	0.59	0.006
Cellular Component (Gene Ontology)				
GO:0043259	laminin-10 complex	3 of 3	2.36	0.00018
GO:0042555	MCM complex	6 of 9	2.18	4.85E-08
GO:0043260	laminin-11 complex	2 of 3	2.18	0.0082
GO:0005606	laminin-1 complex	2 of 3	2.18	0.0082

GO:0042382	Paraspeckles	2 of 6	1.88	0.0188
GO:0002102	Podosome	3 of 29	1.37	0.0145
GO:0022625	Cytosolic large ribosomal subunit	4 of 56	1.21	0.0067
GO:0000784	Nuclear chromosome, telomeric region	6 of 102	1.13	0.0005
GO:0034399	Nuclear periphery	7 of 133	1.08	0.0002
GO:0000781	Chromosome, telomeric region	7 of 134	1.07	0.0002
KEEG pathways				
hsa03030	DNA replication	6 of 36	1.58	9.14E-06
hsa04110	Cell cycle	6 of 120	1.06	0.0032
hsa03010	Ribosome	5 of 130	0.94	0.0355
Reactome pathways				
HSA-176974	Unwinding of DNA	6 of 11	2.09	1.52E-07
HSA-68962	Activation of the pre-replicative complex	6 of 33	1.62	1.33E-05
HSA-176187	Activation of ATR in response to replication stress	6 of 37	1.57	1.71E-05
HSA-3000157	Laminin interactions	4 of 30	1.48	0.0017
HSA-9619665	EGR2 and SOX10-mediated initiation of Schwann cell myelination	3 of 28	1.39	0.0148
HSA-8874081	MET activates PTK2 signaling	3 of 30	1.36	0.0174
HSA-68867	Assembly of the pre-replicative complex	6 of 66	1.32	0.00026
HSA-68949	Orc1 removal from chromatin	6 of 69	1.3	0.00026
HSA-3000178	ECM proteoglycans	5 of 76	1.18	0.0027
HSA-156827	L13a-mediated translational silencing of Ceruloplasmin expression	7 of 108	1.17	0.00026

The proteomes of placentas of women with high and low maternal insulin sensitivity (IS_{HOMA}) were analysed using untargeted proteomics (MS/MS). Proteins enriched (n=86, p<0.05) in the low IS_{HOMA} group ($IS_{HOMA} \leq 0.61$) were selected for network analysis (String V.11.5). The top ten terms for each analysis (biological processes, molecular function, cellular component, KEGG pathways, Reactome pathways) are shown in the table.

Counts in network: the first number indicates how many proteins in this specific network are annotated with a particular term. The second number indicates how many proteins on the human proteome have this term assigned. For example, in this specific network there are 6 out of 7 proteins on the human proteome that have assigned the term ‘pre-replicative complex assembly involved in nuclear cell cycle DNA replication’.

Strength: Log10(observed / expected). This measure describes how large the enrichment effect is. It is the ratio between 1) the number of proteins in this specific network that are annotated with a term and 2) the number of proteins that are expected to be annotated with this term in a random network of the same size.

FDR: False discovery rate. This measure describes how significant the enrichment is. Shown are p-values corrected for multiple testing using the Benjamini–Hochberg procedure.

Supplementary Table 2. Correlation of maternal clinical traits and MCM protein levels.

		MCM2	MCM3	MCM4	MCM5	MCM6	MCM7
Gest Age	r	-0.048	-0.021	-0.140	-0.061	0.004	0.092
	p-value	0.844	0.935	0.631	0.803	0.988	0.717
BMI	r	0.139	0.176	0.057	0.123	0.006	-0.093
	p-value	0.571	0.486	0.847	0.616	0.980	0.714
Leptin	r	0.197	0.262	0.203	0.255	0.098	-0.051
	p-value	0.433	0.308	0.505	0.307	0.699	0.846
Glucose	r	0.119	0.323	0.275	0.316	0.235	0.036
	p-value	0.627	0.191	0.341	0.188	0.333	0.887
C-peptide	r	0.516	0.515	0.569	0.482	0.488	0.379
	p-value	**0.024**	**0.029**	**0.037**	**0.036**	**0.034**	0.121
ISHOMA	r	-0.525	-0.569	-0.560	-0.537	-0.556	-0.406
	p-value	**0.021**	**0.014**	**0.040**	**0.018**	**0.013**	0.095

Correlation calculated by Spearman´s correlation. R: Spearman correlation coefficient. Statistically significant correlations are shown in bold.

Supplementary Table 3. Correlation of MCMs protein levels.

		MCM2	MCM3	MCM4	MCM5	MCM6	MCM7
MCM2	r	1.000	0.653	0.899	0.847	0.835	0.738
	p-value		**0.003**	**<0.001**	**<0.001**	**<0.001**	**<0.001**
MCM3	r	0.653	1.00	0.5	0.849	0.897	0.821
	p-value	**0.003**		0.106	**<0.001**	**<0.001**	**<0.001**
MCM4	r	0.899	0.473	1.000	0.749	0.842	0.819
	p-value	**<0.001**	0.106		**0.003**	**<0.001**	**0.001**
MCM5	r	0.847	0.849	0.749	1.000	0.900	0.839
	p-value	**<0.001**	**<0.001**	**0.003**		**<0.001**	**<0.001**
MCM6	r	0.835	0.897	0.842	0.900	1.000	0.948
	p-value	**<0.001**	**<0.001**	**<0.001**	**<0.001**		**<0.001**
MCM7	r	0.738	0.821	0.819	0.839	0.948	1.000
	p-value	**<0.001**	**<0.001**	**0.001**	**<0.001**	**<0.001**	

Correlation calculated by Spearman´s correlation. R: Spearman correlation coefficient. Statistically significant correlations are shown in bold.

Clinical Traits	BMI < 25 (n=12)	BMI ≥ 25 (n=11)	p-value

	Median; (IQR)	Median; IQR	
Gestational age (days)	45 (35-62)	42 (35-48)	0.379
Maternal age (years)	37 (27-39)	30 (25-36)	0.059
BMI (kg/m2)	22.1 (21.1-22.6)	28.7 (27.4-30.1)	**<0.001**
Leptin (ng/ml)	8.7 (4.1-10.5)	16.3 (13.3-28.9)	**0.002**
Glucose (mmol/l)	4.6 (3.6-5.3)	4.9 (4.7-5.2)	0.169
C-peptide (pmol/l)	313.6 (232.7-365.0)	566.1 (449.6-706.1)	**<0.001**
ISHOMA Index	1.1 (0.73-1.31)	0.47 (0.28-0.57)	**<0.001**

Supplementary Table 4. Maternal clinical traits of the cohort used for immunoblotting.

Maternal clinical traits (median, IQR) of the women of which placenta tissue was subjected to immunoblotting (n = 23) stratified by BMI. Differences were tested by Mann-Whitney U Test. Statistically significant differences are shown in bold.

BMI: Body mass index; IS_{HOMA}: Homeostatic model assessment of insulin sensitivity IQR: Interquartile range

Supplementary Table 5. Maternal clinical traits of the cohort used for immunohistochemistry

Clinical traits	BMI<25 (n=2)<br>Mean (Min-Max)	BMI≥25 (n=2)<br>Mean (Min-Max)
Gestational age (days)	40.5 (35-46)	39.5 (35-44)
Maternal age (years)	30.5 (22-39)	34 (25-43)
BMI (kg/m^2)	20.4 (19.7-21.0)	29.3 (27.4-31.1)
Leptin (ng/ml)	1.8 (1.4-2.1	18.8 (13.3-24.2)
Glucose (mmol/l)	4.0 (3.2-4.8)	5.5 (5.2-5.9)
C-peptide (pmol/l)	322.3 (223.4-322.3)	891.1 (599.2-1183.0)
IS_{HOMA} Index	1.05 (0.93-1.17)	0.28 (0.20-0.35)

Maternal clinical traits (mean; minimum-maximum) stratified by BMI of the women whose placental tissue (n=4) was used for immunohistochemical analysis.

BMI: Body mass index; IS_{HOMA}: Homeostatic model assessment of insulin sensitivity.

Supplementary Figure 1. Allocation of samples to experiments.

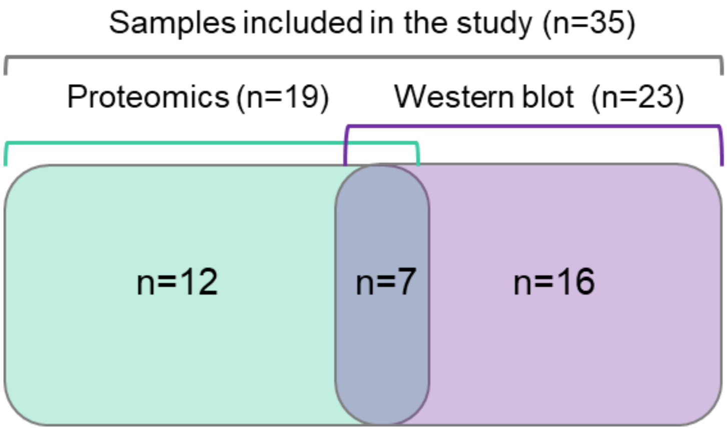

Thirty five first trimester placentas were included in the study. Of these, nineteen were used for proteomics analysis and twenty three for western blotting. Seven samples were used in both types of analyses.

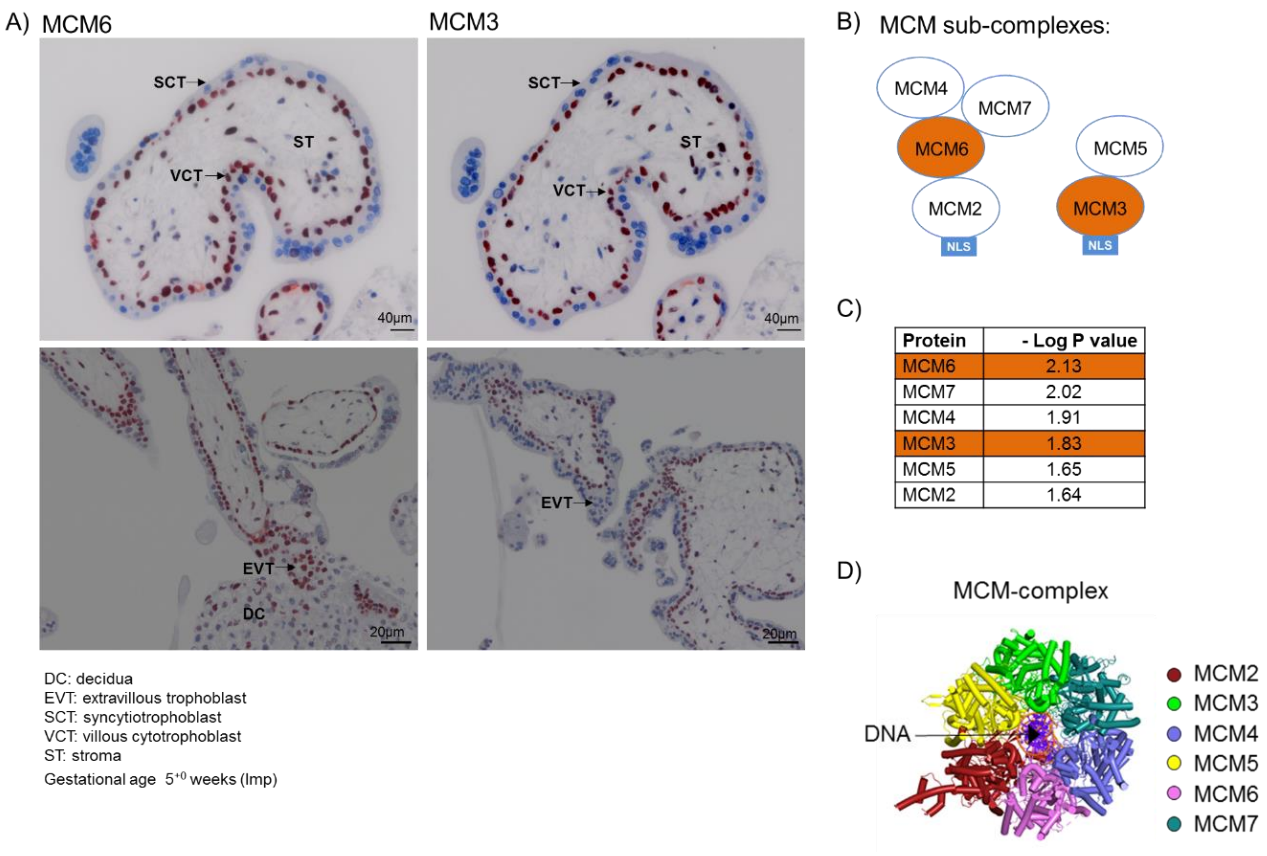

Protein	- Log P value
MCM6	2.13
MCM7	2.02
MCM4	1.91
MCM3	1.83
MCM5	1.65
MCM2	1.64

Supplementary Figure 2. MCM3 and MCM6 are present in the nuclei of villous and extravillous cytotrophoblast cells in the first trimester placenta. A) Placenta sections (gestational age: 6^{+0} weeks LMP) were stained with anti-MCM6 and anti-MCM3 antibodies and nuclei counterstained with Mayer´s hematoxylin. B) The MCM complex is formed by two sub-complexes that are imported separately into the nucleus, where they assemble to form the MCM complex. C) In the proteomics data, MCM6 and MCM3 were the most enriched proteins of each complex. D) PDB structure of the assembled MCM-complex based on identifier 7PLO. Created with PyDock.

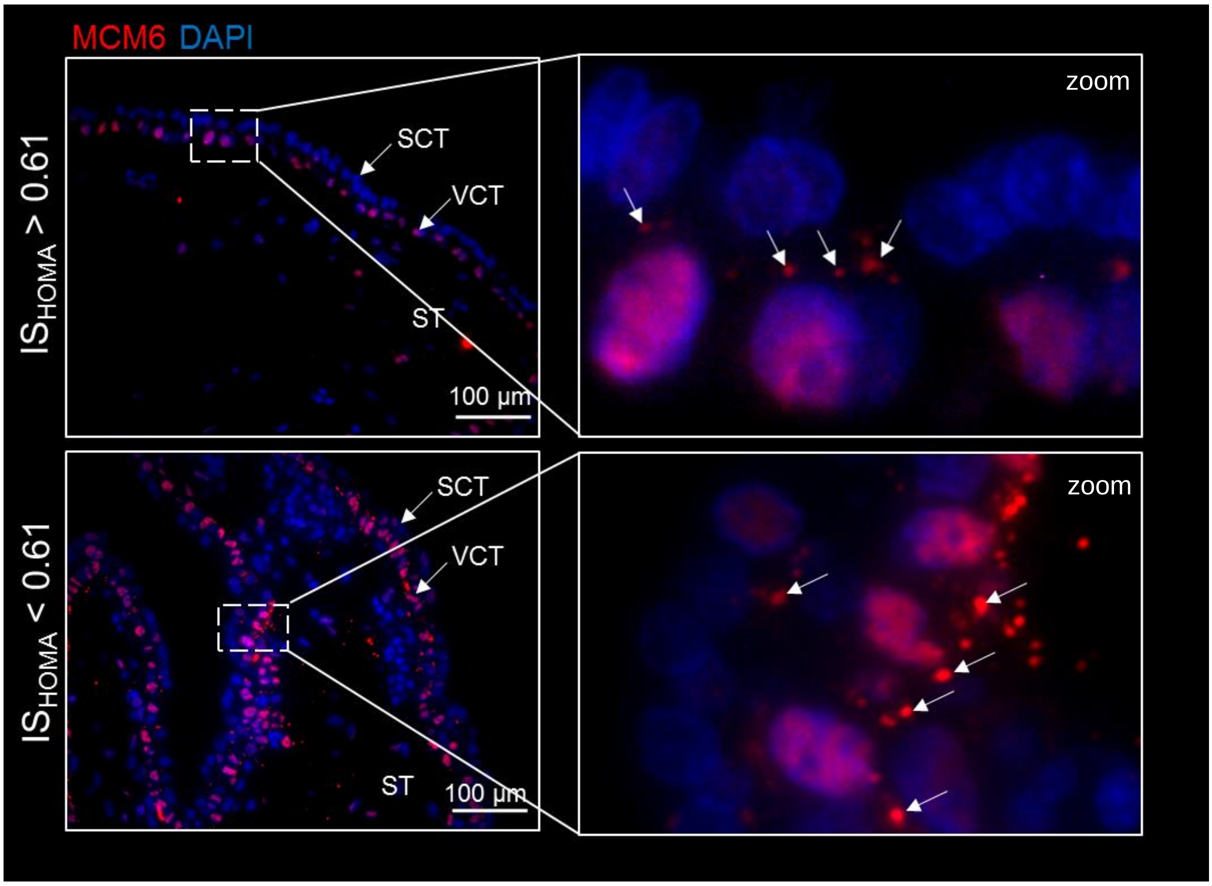

Supplementary Figure 3. MCM6 foci form at the cytoplasm of villous cytotrophoblast cells and the syncytiotrophoblast in the human first trimester placenta.
Placenta tissue of women with high (n=2) and low (n=2) insulin sensitivity was stained with anti-MCM6 antibody and DAPI (nucleus). Representative images are shown. Tissue overview of MCM6 staining acquired in a light microscope (40x). MCM6 localizes predominantly to the nuclei of cytotrophoblast cells, but MCM6 foci are also present in the cytoplasm of villous cytotrophoblast cells and the syncytiotrophoblast. Brightness and contrast adjusted for better visualization.
